# Adult Kawasaki disease in a European patient: a case report and review of the literature

**DOI:** 10.1186/s13256-015-0516-9

**Published:** 2015-04-01

**Authors:** Theano Kontopoulou, Dimitrios Georgios Kontopoulos, Emmanouel Vaidakis, George P Mousoulis

**Affiliations:** 1 3rd Department of Internal Medicine, Evangelismos Hospital, Ipsilantou 45-47, 10676 Athens, Greece; 2 Department of Life Sciences, Imperial College London, Silwood Park Campus, Buckhurst Road, Ascot, SL5 7PY UK; 3 Department of Internal Medicine, Metropolitan Hospital, Ethnarchou Makariou 9 & El. Venizelou 1, 18547 N. Faliro, Athens Greece

**Keywords:** Kawasaki disease, Mucocutaneous lymph node syndrome, Vasculitis

## Abstract

**Introduction:**

Kawasaki disease is an acute necrotising vasculitis of the medium- and small-sized vessels, occurring mainly in Japanese and Korean babies and children, aged 6 months to 5 years. Its main complication is damage of coronary arteries, which has the potential to be fatal. Here we report a rare case of Kawasaki disease that occurred in a 20-year-old Greek adult.

**Case presentation:**

A 20-year-old Greek man presented with high fever, appetite loss, nausea and vomiting, headache and significant malaise. He had an erythema of the palms and strikingly red lips and conjunctiva. As he did not respond to broad-spectrum antibiotics and after having excluded other possible diagnoses, the diagnosis of Kawasaki disease was set. He was treated with intravenous immunoglobulin and oral aspirin on the 10th day since the onset of the illness. His clinico-laboratory response was excellent and no coronary artery aneurysms were detected in coronary artery computed tomography performed 1 month later.

**Conclusions:**

This report of an adult case of European Kawasaki disease may be of benefit to physicians of various specialties, including primary care doctors, hospital internists, intensivists and cardiologists. It demonstrates that a case of prolonged fever, unresponsive to antibiotics, in the absence of other diagnoses may be an incident of Kawasaki disease. It is worth stressing that such a diagnosis should be considered, even if the patient is adult and not of Asian lineage.

## Introduction

**Figure 1:**
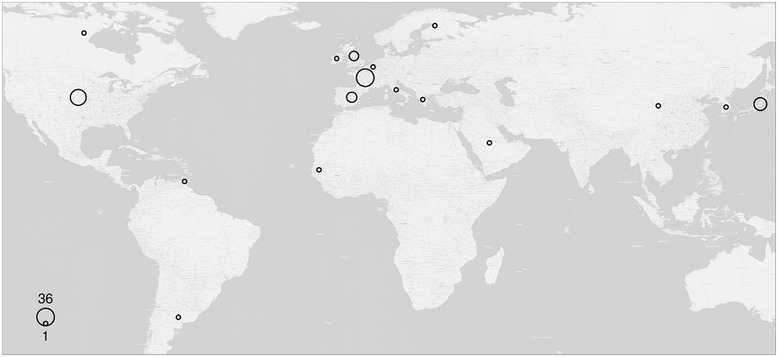
**A global map of reported adult Kawasaki disease cases, including the present one.** The size of the circles is proportional to the number of published cases in each country, ranging from 1 to 36.

Kawasaki disease (KD), also known as mucocutaneous lymph node syndrome, is an acute necrotising vasculitis of the medium- and small-sized vessels. It was first described by Tomisaki Kawasaki in 1967. It occurs most often in babies and children, aged 6 months to 5 years and the male-to-female ratio ranges from 1.5–1.8 to 1. KD is most prevalent in Japan, while Korea holds the second place as to the number of patients. Its incidence in Japanese and Korean children living in the USA and following a Western lifestyle is higher than in Caucasian children. Since the disease is not common in adults, especially in Europe, it is very often misdiagnosed [1-4]. According to a review in 2011, 91 cases of adult KD had been described until that time [5]. Eight more cases have been added to the medical literature since then [6-13]. To the best of our knowledge, our case is the 100th one. It is the first described adult Kawasaki case not only in Greece, but also in South-Eastern Europe (Figure 1).

### Pathogenesis and genetics

The pathogenesis of KD is to a very large extent unknown. A passive protection by maternal antibodies could possibly explain the rarity of the illness during the first months of life of an individual. An infectious cause might be possible, since seasonal outbreaks, national epidemics, associations with tropospheric wind patterns [14] and a self-limited course of the disease have been observed [15]. Some studies point out that the trigger for KD could be a bacterial superantigen [2,3] or a fungal toxin [16]. The immune character is suggested by B and T cells activation and cytokines production. Moreover, the severity of KD symptoms has been correlated with the expression of *THRIL*, a long noncoding ribonucleic acid involved in tumour necrosis factor (TNF) α regulation [17]. Also of interest, a murine model of KD develops a similar coronary arteritis after a single intraperitoneal injection of group B *Lactobacillus casei* cell wall extract [18].

A genetic predisposition hypothesis is supported by: i) noticeable differences in incidence rates among people of various ethnic groups along with ii) a higher incidence of KD among siblings of a patient compared to the general population. Nevertheless, the genetic background of KD remains mostly unclear. A series of polymorphisms in a number of genes have been linked with increased susceptibility for KD, with the most notable ones being in *ITPKC* [19] and *CASP3* [20]. Finally, the genetic loci of *FCGR2A*, *HLA*, *BLK* and *CD40* have also been suggested as susceptibility candidates by means of genome-wide association studies [21-23].

### Diagnosis and complications

KD diagnosis is absolutely clinical since no specific diagnostic tests are available [5], although the proteins meprin A and filamin C were recently shown to possess potential as KD biomarkers [24]. The diagnosis is possible if an individual has persistent fever for more than 5 days, is unresponsive to broad-spectrum antibiotics, has no proof of infection and four out of the five following criteria: i) polymorphous rash; ii) conjunctivitis; iii) cervical lymphadenopathy; iv) oral changes including injected pharynx or lips, cracked or fissured lips, strawberry tongue; v) extremity changes starting with oedema or erythema and then progressing to desquamation of feet and hands, starting periungually [1,2,12,15]. Unfortunately, these diagnostic criteria are valid for the diagnosis of KD in children, but have not yet been validated in adults [5].

Coronary artery damage is the main and sometimes fatal complication of the disease, being expressed as aneurysm, calcification or stenosis [25,26]. Coronary artery aneurysms (CAA) can change with time: they may regress, stay unchanged, progress to obstruction and, rarely, rupture or expand [3]. If coronary abnormalities are present, the diagnosis of KD can be set even when less than four of the aforementioned criteria are met [5,27]. Susceptibility to coronary artery lesions formation has been associated in various populations with specific single nucleotide polymorphisms in a wide range of genes, such as *ITPKC* [19], *IL-10* [28], *TGFBR2* [29], *DC-SIGN* [30] and *KCNN2* [31]. Arteries other than the coronary ones are also affected by the disease: a hepatic artery aneurysm [32], a left humeral artery aneurysm, with absence of flow in the antebrachial arteries causing a peripheral gangrene [33], a proximal gastroduodenal artery occlusion, and a mildly irregular splenic artery associated with obstruction of the distal intrasplenic branches [34] are described in the literature.

Systemic inflammation of many organs can occur. Inflammation of the central nervous system would cause typical meningitis [35] or just headache [2,8,32,36-43]. As to the myocardium and pericardium, KD may be the cause of tricuspid regurgitation with vegetations [44], mitral valve regurgitation with thickened mitral leaflets [45], pericardial effusion [45], myocarditis [12,36] or heart failure [12,46,47].

Clinical and laboratory findings concerning liver inflammation are jaundice [32,40,48,49], hepatomegaly [15,32,50], right upper quadrant pain [32,39], cholestatic hepatitis [6,44,46-49,51], hepatic artery aneurysm [32], elevated aspartate and alanine aminotransferase levels [2,8,11,13,33,34,40,41,43,52,53] and low albumin levels [11,40,53].

Mild renal failure, dysuria [6], as well as sterile leucocytosis in urine analysis [11,15] can be manifestations of renal involvement in KD.

The upper and lower respiratory system may also be affected. Reddened pharynx [35,38-43,47-49,54], sore throat [2,36,39,41,43,44,51,55-58], rhinorrhoea [43], mild non-productive cough [39,43,44], dyspnoea [46], laboured respirations [32,48], rales [47], and acute respiratory distress [47] are symptoms often encountered.

The upper and lower intestinal tract are involved in KD as well. Nausea [2,6,32,37,39,41,43,48,49,55,56,58], vomiting [6,35,37,39-41,43,44,55], stomach cramps [48], epigastric pain [47,49], dysphagia [34,40,51], abdominal pain [39,40,51,58], abdominal tenderness [49], abdominal distension [47], and diarrhoea [32,39-41,47-49] are the main symptoms and signs.

The musculoskeletal system participates in the inflammatory process with various clinical representations: arthralgia of the big joints [2,34,40-43,46,48,49,51,55,58] or of the small ones [57], arthritis of the big joints [42], back pain [39], hand and foot pain [39,41], painful synovial thickening without effusion [32], joint effusion [2], joint pain with or without motion [2,44], joints tender to palpation [51], and myalgia [27,36,43,49,51,53].

Lymphadenopathy is often observed in KD. It may be mainly cervical [2,6,8,10,12,13,32-34,36,37,39,40,42,44,46-52,55-58], but also axillary [33,36,40], inguinal [40,59] or diffuse [38,41,43,45,54].

Skin and mucous membranes express a variety of abnormalities: skin rash [2,8,12,13,15,33-36,38-50,52-56,58]; skin eruptions [12,36,41,51]; polymorphic erythema [6,10]; erythema of palms and soles [15,34,40-42,45,48,51,54,55]; perineal erythema [12,35,36,40,52]; induration and swelling of hands and feet [6,8,12,13,34,36,39-43,46,51,56,58]; desquamation of palms and soles [2,10,11,13,32-35,37,38,40-47,49,57,59]; strawberry [10,13,32,35-37,40,41,44-48,50-52,55,57,59], fissured [2] or coated tongue [15,48]; angular stomatitis [12,35,40,44,52]; mucosal ulcers [51,56]; erythematous [36,42,45,50,51,57], fissured [40-42,48,49] or oedematous [8,11] lips; erythematous mucous membranes [32,38]; mucositis of the urethra without discharge [41]; and icterus [47,49].

Eye problems are frequent and of great variety: conjunctivitis [2,8,10-13,32-34,36-52,54,55,57-59]; iritis [37]; keratitis [34,52]; icteric sclera [32,47]; painful eyes [37]; palpebral oedema of the eyes [40]; and reduction of vision [52]. As to the ear, tinnitus and mild bilateral hearing loss have been reported [34].

Adult KD seems to occur more frequently in patients with human immunodeficiency virus [5,37-40,42,50,58]. Diseases to be differentiated from Kawasaki are viral and bacterial infections, drug hypersensitivity syndromes, toxic shock syndrome and autoimmune diseases with idiopathic juvenile arthritis being a representative example [2,5,25,27,60].

### Treatment and long-term outcome

Ideal treatment for KD should be immediate administration, no later than on the 10th day of the disease onset, of both immunoglobulin at 2g/kg once and aspirin at 50 to 100mg/kg for as long as the inflammation is present [6,8,39,40,45,49,52,56]. Unfortunately, diagnosis within 10 days since the disease onset is set only in very few cases [6,8,13,36,45,49,52], whereas treatment is given late in others [2,15,37,44,50,51,55-57]. In a review in 2011, only 15 out of 91 patients with KD were administered treatment with both intravenous immunoglobulin (IVIG) and aspirin within the first 10 days of the disease onset [5]. Some patients received only aspirin [15,35,36,43,44,46-48,51,55,57], while others were treated with IVIG only [37,40,50,51].

Individuals who do not respond to the dual treatment [2,3,6,12,42,61] have refractory KD and should be treated with a second dose of immunoglobulin (2g/kg) [39,42] and in non-responsiveness with high doses of corticosteroids, tapered progressively until C-reactive protein (CRP) regresses to normal levels [62]. A third [42] and fourth [34] dose of immunoglobulin have also been reported for relapses. Immunosuppressive factors, such as anti-TNF, could be a potentially beneficial treatment [3,26,27,63,64]. Treatment choices in refractory KD could also be plasmapheresis and thalidomide [42].

The long-term outcome of adults with a history of KD without CAA is excellent [2,5,13,15,34,36,39,40,43,44,46-49,55,56]. Anticoagulation treatment should be discontinued after 6 to 8 weeks and a healthy lifestyle should be adopted [26]. By contrast, patients with large aneurysms (>6mm), regressed large ones or persistent small ones are at high risk for developing stenosis or ischemia. They should be put on strong anticoagulation therapy and lifelong follow-up [2,3,7,9,10,33,53,65]. These individuals have to be periodically tested with echocardiography, stress tests, computed tomography angiography or magnetic resonance imaging. Catheter-based angiography, although invasive, may sometimes prove useful [5].

## Case presentation

A 20-year-old Greek man presented to our hospital in late May of 2010 because of high fever (up to 40°C, unresponsive to anti-inflammatory drugs), appetite loss, nausea and vomiting, persistent headache and a feeling of significant malaise for 5 days. He was diagnosed at a provincial hospital with an atypical infection and had received azithromycin for 3 days, without any improvement.

He had not travelled recently either within the country or abroad. He denied intravenous drug use, new sexual partners or tattoos. He had no animal exposure. He did not use tobacco products or alcohol and had not taken any medication. He was previously healthy. According to his medical history, he reported allergic rhinitis and conjunctivitis for his first 5 years of life, chickenpox at the age of 5 years, a streptococcal pneumonia at the age of 6 years, several episodes of tonsillitis until the age of 12 years and a tonsillar abscess at the age of 11 years. He reported no drug allergies. No contacts with similar symptoms were identified. He had no antecedents of Asian origin. The family history was non-contributory and no family members had rheumatic diseases.

On admission, his temperature was 38.3°C, blood pressure at 80/50mmHg, pulse was measured at 100 beats per minute and blood oxygen saturation at 92%. A physical examination revealed bilateral conjunctival chemosis, strawberry lips and tongue, dry mucus membranes, a mild skin rash of his trunk, erythema of his thenar and opisthenar regions of both palms, an oedema of palms and soles and tachycardia with S3 and S4 gallop. Results of examinations of his lungs, abdomen, neurological and musculoskeletal systems were normal.

**Figure 2:**
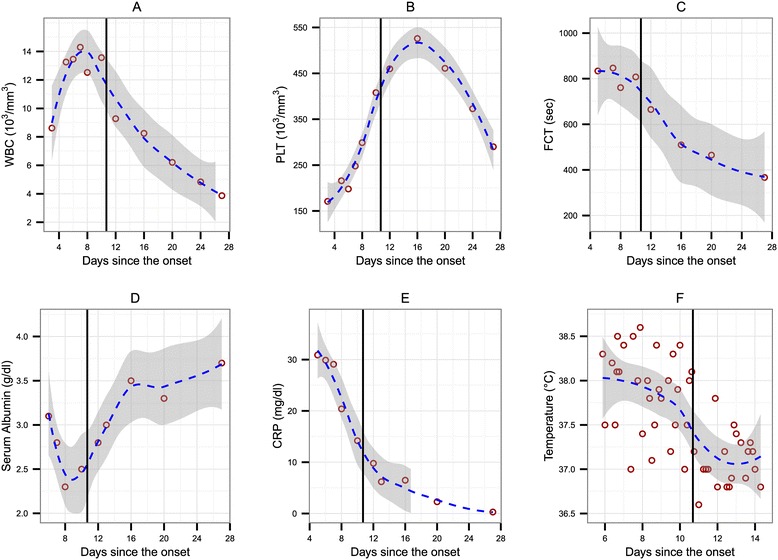
**Evolution of clinico-laboratory data before and after intravenous immunoglobulin administration (A-F).** Open circles correspond to the measured values of the patient. The general time trend for each variable is depicted via fitted Loess curves (in blue), with grey areas showing the 95% confidence interval. The black vertical line indicates the intravenous immunoglobulin treatment event. CRP, C-reactive protein; FCT, Fibrinogen clotting time; PLT, Platelets; WBC, White blood cell count.

Laboratory findings (Figure 2A-F; see also the supporting data set [66]) revealed leucocytosis (13.2×10^3^ per cubic millimetre) with neutrophilia (91%) and left shift with segmented neutrophils, anaemia (haematocrit 33%) and normal platelets upon admission, which were later elevated (Figure 2B). Acute phase reactants were elevated: erythrocyte sedimentation rate (ESR), 127mm 1st hour; CRP, 30mg/dL and fibrinogen 761 seconds. Serum creatinine levels and serum glutamic oxaloacetic transaminase rates were normal, serum glutamic-pyruvic transaminase at 80IU/L, alkaline phosphatase at 147IU/L, gamma-glutamyl transpeptidase at 67IU/L, lactate dehydrogenase at 263IU/L, creatine kinase at 254IU/L, bilirubin was normal, albumin at 3.1g/dL and sodium at 132mmol/L. Urine analysis was normal. Results of blood and urine bacterial cultures and serological tests for bacteria and viruses were negative. Alpha 2 and gamma immunoglobulin levels were elevated. All blood tests for autoimmune diseases were negative. Immunophenotype examination revealed reduced cardinal number of CD3+CD8+ T lymphocytes. The results of a chest X-ray, an electrocardiogram and a transthoracic echocardiogram were normal.

The patient did not respond to broad-spectrum antibiotic treatment. Desquamation of his fingers and toes began on the seventh day since the disease onset. Autoimmune diseases and juvenile idiopathic arthritis were excluded, as he did not fulfil diagnostic criteria for any of these diseases. Drug hypersensitivity reactions could explain his clinical appearance; however, he was previously healthy and there was no need for him to take medication. Immediately after the suspicion of KD (on the 10th day after the onset of symptoms), he was given IVIG at 2000mg/kg once and aspirin at 50mg/kg orally for the first 3 days, and at 100mg per day for another 3 months. His treatment was based on the diagnostic criteria developed for KD in children because there had been no validated criteria for adult cases of KD. Antibiotic treatment was discontinued at this time.

Immunoglobulin and aspirin had a striking result, since the patient was afebrile and in better condition on the very next day after the initiation of treatment. During the following days, all clinico-laboratory findings gradually improved and only a reactive thrombocytosis remained. In total, he remained hospitalised for 10 days. A computed tomography coronary angiography performed 1 month later showed no coronary aneurysms. Today, about 5 years later, our patient remains healthy.

## Discussion

Our patient developed the disease in late May. Thorough investigation for many infectious agents did not prove an infectious source of the disease [2]. Nevertheless, he was administered broad-spectrum antibiotics for 7 days. This treatment was not successful and revealed once more that an infection was absent, at least during the patient's hospitalisation. Our differential diagnosis covered a wide range of diseases, mainly infective and autoimmune; none of them could be proved. Given that his condition deteriorated and having no obvious diagnosis available, we performed an extensive review of the current literature, focusing on his symptoms. KD, although rare in adults, could explain the entirety of symptoms and represent a diagnosis of exclusion. Treatment began immediately on the 10th day of the illness, due to suspicion of KD. This was fortunately in contrast to the vast majority of reported adult cases of KD who received treatment long after the 10-day period [5]. In any case, the treatment that was administered would have caused no harm to the patient, even if KD was not present. It is worth noting that even with adequate and timely administration – within ten days – of IVIG and acetylsalicylic acid (ASA), 5% of patients develop coronary abnormalities [26,62]. The therapeutic result of IVIG supported the diagnosis of KD, since our patient’s condition improved considerably within hours.

Our patient did not have some signs and symptoms that are more usual in adult KD, compared to that in children, such as lymphadenopathy and articular involvement. Lymphadenopathy is present in 93% of adult cases of KD, compared to 75% of child cases. Joint problems are observed in 63% of adults with KD, versus 24 to 38% of children with the disease according to a review in 2011 [9].

In contrast, our adult patient had persistent headache that subsided only after the administration of IVIG and ASA. Involvement of the central nervous system takes place more often in children with KD (34%) than in adults (10%) [5].

Our patient’s response to the treatment was astonishing and extremely quick: fever resolved and his general condition ameliorated within hours. Such a response is not usual in the KD literature [54,39,40,56]. Most of the patients become afebrile and feel better after 2 days [6,8,33,44,45,51,52]; some others after 1 [13,35,39,59] or 3 days [11,50,58].

Laboratory findings consistent with KD were leucocytosis with neutrophilia, anaemia, late thrombocytosis, elevated ESR and CRP, hypoalbuminemia, hyponatremia, elevated serum transaminases and gamma-glutamyl transpeptidase [27].

Other than a single IVIG administration and high doses of ASA during the acute phase, no supplementary IVIG or even corticosteroids or immunosuppressive agents were given, since no symptoms persisted or relapsed [5,62]. ASA was continued for 3 months after the acute phase as an antiplatelet agent [26]. However, some studies suggest immediate discontinuation of all medications after the acute phase, in the absence of CAA [62]. Finally, long-term follow-up was not needed, as no CAA were developed, classifying the patient as risk level I [62]. He was only advised of counselling every 5 years [26].

## Conclusions

This case makes the point that a prolonged fever without response to antibiotics should raise the physician's suspicion of a possible KD incident, considering though its rare occurrences in European adults. Prompt diagnosis and timely treatment of this potentially fatal disease are the keys that can save lives, preventing CAA formation.

Physicians of various specialties are involved either in recognition or treatment of KD. Primary care doctors, being the first to come across the patients, should transfer them to hospitals at once. Hospital internists should then set the diagnosis, according to the relevant criteria, having first excluded other possible diseases. It is vital that patients receive treatment with IVIG and ASA as soon as possible and no later than 10 days since the onset of symptoms. Cardiac complications and hemodynamic instability may bring the need for transfer to an intensive care unit, relegating treatment to intensive care practitioners. Finally, cardiologists have the responsibility of tracing long-term follow-ups, along with monitoring any arising cardiac issues.

Future studies will hopefully shed light on the origins and aetiology of the disease, allowing for more efficient and targeted treatment and follow-up.

### Availability of supporting data

The data set supporting the results of this article is available in the Figshare repository [66].

## Consent

Written informed consent was obtained from the patient for publication of this case report and accompanying images. A copy of the written consent is available for review by the Editor-in-Chief of this journal.

## Abbreviations

ASA: Acetylsalicylic acid
CAA: Coronary artery aneurysms
CRP: C-reactive protein
ESR: Erythrocyte sedimentation rate
IVIG: Intravenous immunoglobulin
KD: Kawasaki disease
*THRIL*: TNFα and heterogenous nuclear ribonucleoprotein L related immunoregulatory large intergenic noncoding ribonucleic acid
TNF: Tumour necrosis factor
